# Production and Characterization of Biodegradable Wound Dressings Containing Silver-Loaded Zeolite Complexes

**DOI:** 10.1021/acsomega.4c10481

**Published:** 2025-03-20

**Authors:** Aline Carvalho Lopes, Ana Beatriz Klosowski, Juliana Bonametti Olivato

**Affiliations:** Pharmaceutical Sciences Department, State University of Ponta Grossa, 84030-900 Ponta Grossa, Paraná, Brazil

## Abstract

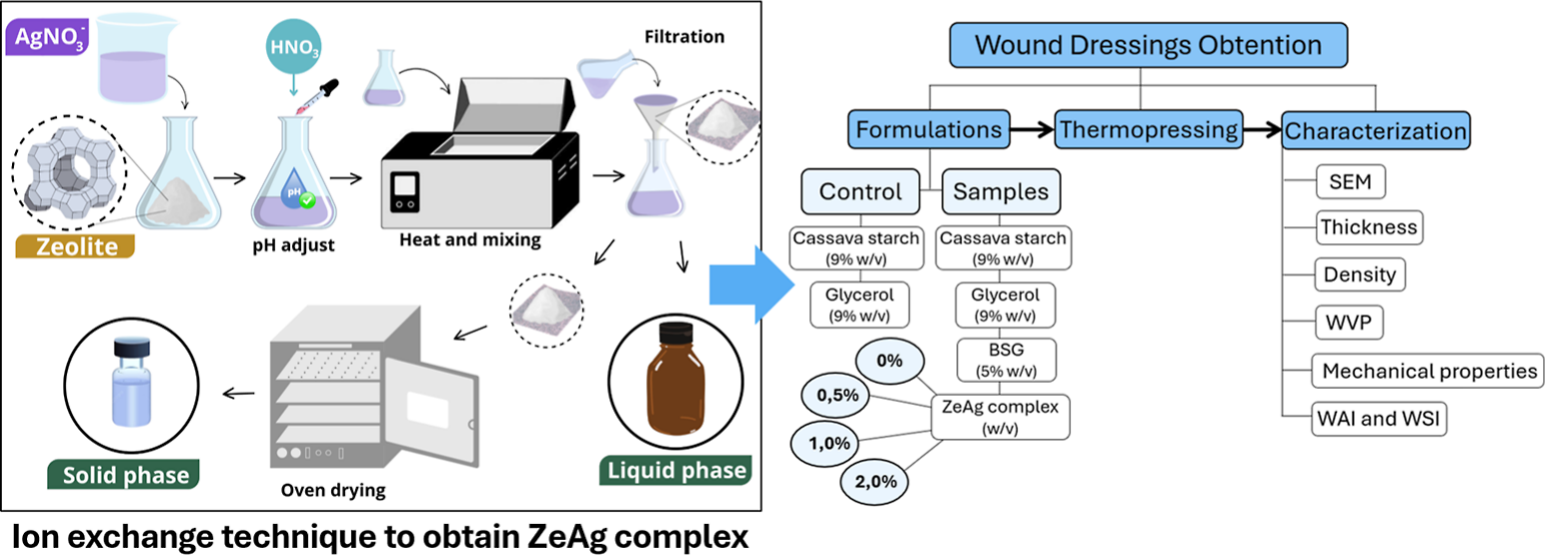

Silver-loaded zeolite complexes (ZeAg) were incorporated into biodegradable wound dressings based on starch and brewers’ spent grain. Four formulations (0, 0.5, 1, and 2% (w/v) of ZeAg) of wound dressings containing ZeAg were produced by thermopressing, and their morphology and structural and mechanical properties were evaluated. The dressings showed a rough and porous surface structure, and no significant differences were observed in the thickness, density, solubility, or water vapor permeability of the samples. The dressing with the lowest concentration of ZeAg (0.5%) demonstrated enhanced water absorption, while the sample with the highest concentration (2.0%) showed improved mechanical properties, including greater tensile strength, elongation at break, and elastic modulus. According to these findings, incorporating ZeAg complexes into starch-based wound dressings can improve their functional properties, which makes them promising candidates for advanced wound care applications.

## 1 Introduction

Biopolymers of natural sources have gained significant attention due to their characteristics of biodegradability, biocompatibility, nontoxicity, processability, and availability.^1,2^ Starch-based biopolymers are a notable category, but they have drawbacks such as high water vapor permeability (WVP) and low mechanical performance, resulting in brittle materials. To overcome these challenges, lignocellulosic fibers have emerged as a promising alternative for reinforcing starch-based polymeric materials.^3−5^

Lignocellulosic fibers are renewable raw materials from natural sources and agro-industrial wastes.^6^ The brewing industry produces a significant quantity of lignocellulosic material called brewers’ spent grain (BSG), which corresponds to approximately 85% of the total mass of solid byproducts of the brewing industry. Wet BSG production is typically 20 kg for every 100 L of beer.^7,8^ The BSG is biodegradable and primarily used as animal feed and biofuel and in the food industry. Its rich composition is attracting attention as a sustainable alternative to petrochemical resources when producing fuels, plastics, and high-value biomaterials.^9,10^

Biocompatible and biodegradable, the BSG can be a promising reinforcing agent for polymeric materials in biomedical applications.^11^ Lignocellulosic materials, such as the BSG, can be utilized in a variety of medical devices, including prosthetics, implants, wound dressings, and drug delivery systems.^12,13^ Innovation, technological advancement, and sustainable resource utilization are all possible outcomes from the development of biomaterials that incorporate the BSG, which supports the principles of the circular economy by promoting the efficient use of byproducts.

Biodegradable wound dressings are a common use of natural polymers.^14^ Wound dressings provide protective barriers during the healing process, a complex biological process that involves a variety of cell types, growth factors, and extracellular matrix components.^15,16^ Molecular mechanisms of wound healing have been extensively studied, with emphasis on the contributions of fibroblasts, keratinocytes, and macrophages to tissue repair.^17^ Biomaterials, including hydrogels, dressings, and bioactive compounds, have demonstrated their potential to improve the wound healing process by creating a favorable microenvironment for cellular activity.^18,19^

Antimicrobial dressings are highly effective at managing infected and exudative wounds. Their high porosity permits excellent absorption capacity and prevents microbial contamination at the wound site.^20^ Among antimicrobial agents, silver compounds (Ag^+^) are well-known for their efficacy against resistant microorganisms.^21,22^ Silver has a broad-spectrum antimicrobial activity at low concentrations with relatively low toxicity to humans, making it suitable for biomedical use.^23^ Silver has beneficial antibacterial effects that are well-characterized, but the proposed mechanisms of action differ greatly. The mechanisms of action of silver include the generation of reactive oxygen species, cell membrane damage, inhibition of respiration, and inactivation of iron–sulfur clusters of bacterial dehydratases involved in amino acid biosynthesis.^24,25^ The uncontrolled release of Ag^+^ ions limits its effectiveness and safety, which can be addressed using carrier materials, such as zeolites.^26,27^

Zeolites are natural or synthetic microporous minerals composed of silicon and aluminum molecules.^28,29^ Ion-exchange capacity and thermal stability of zeolites make them suitable carriers for the controlled release of active agents, including silver ions.^30,31^ Combining zeolite with silver allows for controlled antimicrobial activity while enhancing the durability of the material,^32^ also more effective healing with reduced risk of resistance development can be achieved.^33,34^

In this context, silver-loaded zeolites combined with BSG-reinforced starch-based polymeric materials offer a novel strategy for producing biodegradable wound dressings. Therefore, the aim of this study was to produce biodegradable wound dressings based on starch and BSG with different proportions of silver-loaded zeolite complexes. The dressings were evaluated for their structural, barrier, and mechanical properties, which provides an alternative for developing biodegradable polymers for biomedical applications.

## 2 Materials and Methods

Cassava starch was obtained from Agrícola Horizonte Group (Paraná, Brazil), glycerol was purchased from Reagen Produtos para Laboratório LTDA (Paraná, Brazil), the lignocellulosic fibers used in this research were supplied by “Koch Beer” Microbrewery (Paraná, Brazil), the silver nitrate (AgNO_3_) P. A. was obtained from Rea-Tech (Paraná, Brazil), and the zeolite Y (crystalline aluminosilicate; powder; pore size < 10 Å, Si/Al ratio 2.547 ± 0.037) was purchased from Sigma-Aldrich (USA).

### 2.1 Silver-Loaded Zeolite Complexes Production

**Figure 1:**
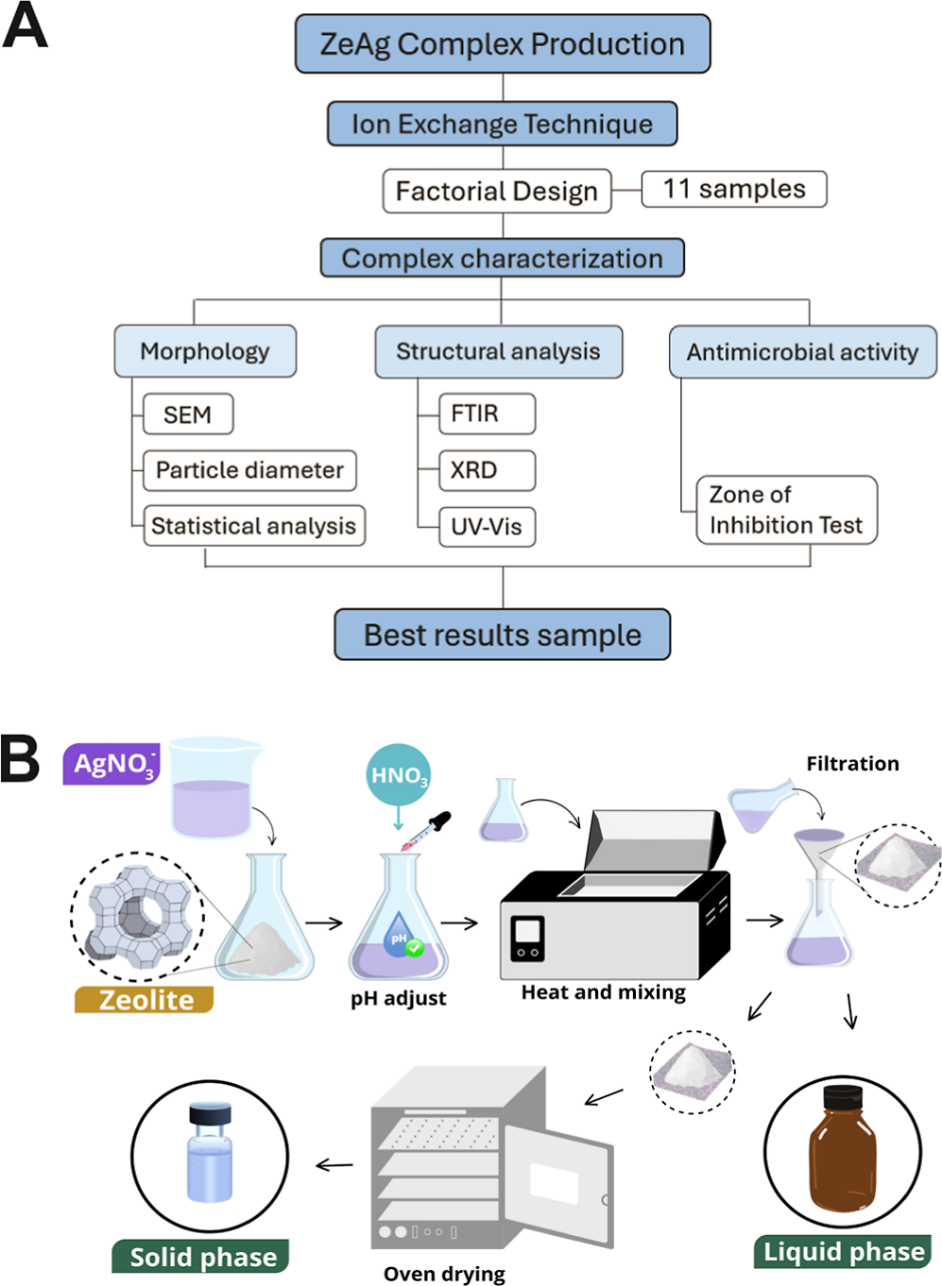
(A) Diagram of zeolite silver-loaded (ZeAg) production. (B) Ion-exchange technique to obtain the ZeAg complex.

The silver-loaded zeolite (ZeAg) complex was produced with pH 5,0, a temperature of 45 °C, and a AgNO_3_ concentration of 4,000 mg/L using the ion-exchange impregnation technique, as described by Lopes et al.^35^ (Figure 1).

### 2.2 Wound Dressings Obtention

**Figure 2:**
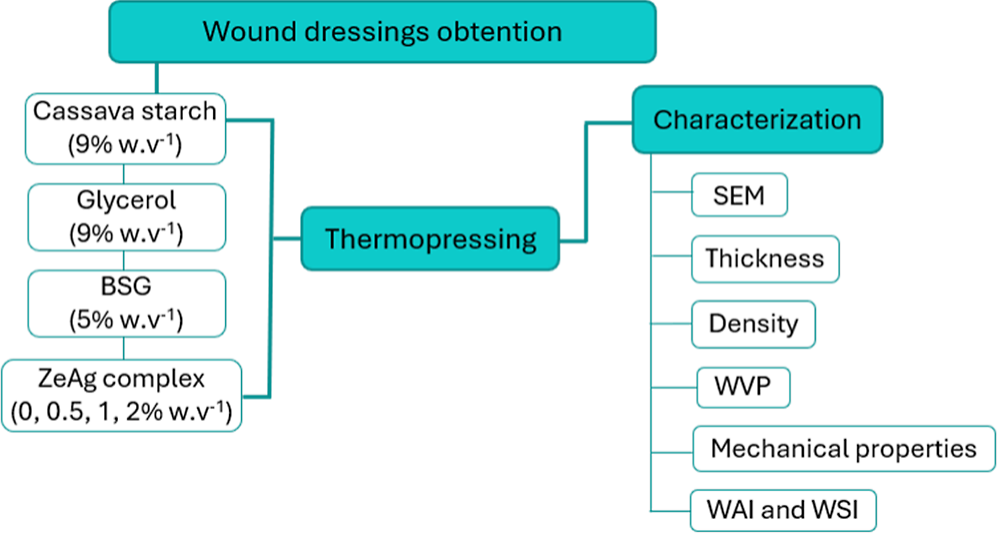
Diagram of wound dressings obtention.

Four samples were tested to obtain the wound dressings by the thermopressing method (Figure 2). The components of the formulation included cassava starch (85% w·v^–1^), glycerol (9% w·v^–1^), BSG (5% w·v^–1^), and different concentrations of the ZeAg complexes (0, 0.5, 1, and 2% w·v^–1^). These components were mixed in 100 mL of water and manually stirred. Subsequently, the mixtures were weighed, obtaining approximately 100 g of each formulation, and this amount was transferred to the previously heated thermopress (175 °C/1 atm), for 18 min. The formulations were stored at 25 ± 2 °C until the analysis.

### 2.3 Scanning Electron Microscopy Analysis

A Tescan model Mira 3 scanning electron microscope was used to make observations on the surface of the dressings. Before coating with a gold layer, the samples were stored at 25 ± 2 °C in a desiccator with CaCl_2_ (0% RH) for 7 days. The coating was performed with a Sputter Coater (Quorum SC7620). Images were taken of the surface, with 1000× of magnification.

### 2.4 Thickness and Density of Wound Dressings

The thickness of the samples was measured with a digital micrometer with resolution 0.001 mm, through 5 random points. For the density, the samples (2 cm × 2 cm) were stored at 25 °C in a desiccator with CaCl_2_ (0% RH) for 7 days and then weighed to determine the weight of samples. The thickness, width, and length were evaluated to perform the density calculation.

### 2.5 Water Solubility and Water Vapor Permeability

For the water solubility test, the samples were previously dried in a desiccator containing CaCl_2_ (0% RH) for 7 days. After this period, they were weighed and immersed in distilled water (30:1 ratio—water:sample) for 48 h at 25 °C. Excess water was removed and the material was dried in an oven at 105 °C until a constant mass, which is used as the final mass of the samples to calculate the water solubility of the materials. The determinations were performed in triplicate. The WVP was determined by the gravimetric method with adaptations from the American Society for Testing and Material (E96-95).^35^

### 2.6 Water Absorption and Water Solubility Indexes

The water absorption index (WAI) and the water solubility index (WSI) were determined according to Rani et al.^36^ with some modifications. Approximately 2.5 g of sample and 30 mL of water were placed in a centrifuge tube and previously weighed. A mechanical shaker was used to shake the tubes for 30 min, and then they were centrifuged at 3000 rpm for 5 min. The supernatant liquid was carefully transferred to a Petri dish and placed in an oven at 105 °C until it reached a constant weight. The remaining material from the centrifuge tube (gel) was weighed and the WAI (g water/g solid) was calculated according to eq 1 and WSI in eq 2
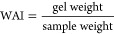
1
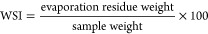
2

### 2.7 Mechanical Properties

A universal mechanical testing machine (Shimadzu, Kyoto, Japan) was used to determine the tensile properties of the dressings. Tensile tests were based on American Society for Testing and Material Standard (ASTM) D882-91,^35^ by using 50 × 20 mm samples and a speed test of 50 mm·min^–1^. The tensile strength (MPa), elongation at break (%), and Young’s modulus (MPa) were determined.

### 2.8 Statistical Analysis

The data were expressed as mean and standard deviation, followed by ANOVA analysis and Tukey’s test, with 5% variance (*p* < 0.05).

## 3 Results and Discussion

### 3.1 Wound Dressings Production

**Figure 3:**
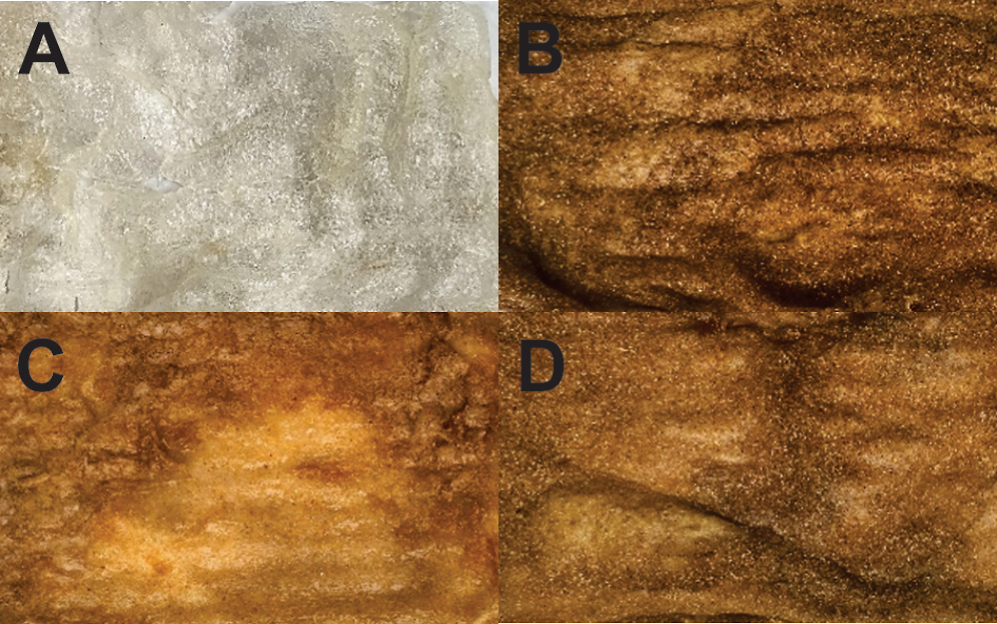
Visual appearance of the wound dressings.

Figure 3 shows the visual characteristics of the wound dressing formulations. The brownish color is caused by the inclusion of 5% BSG in the formulations (Figure 3B–D). The materials demonstrated a highly porous surface as well as malleability and mechanical resistance, which made them easy to handle without any visible damage. The coloration of extruded starch-based composites containing BSG was affected by higher fiber content, as reported by Lopes et al.^37^ The BSG proportion was constant in all tested formulations in this study, which resulted in no noticeable differences in coloration among samples containing lignocellulosic fibers.

### 3.2 Scanning Electron Microscopy Analysis

**Figure 4:**
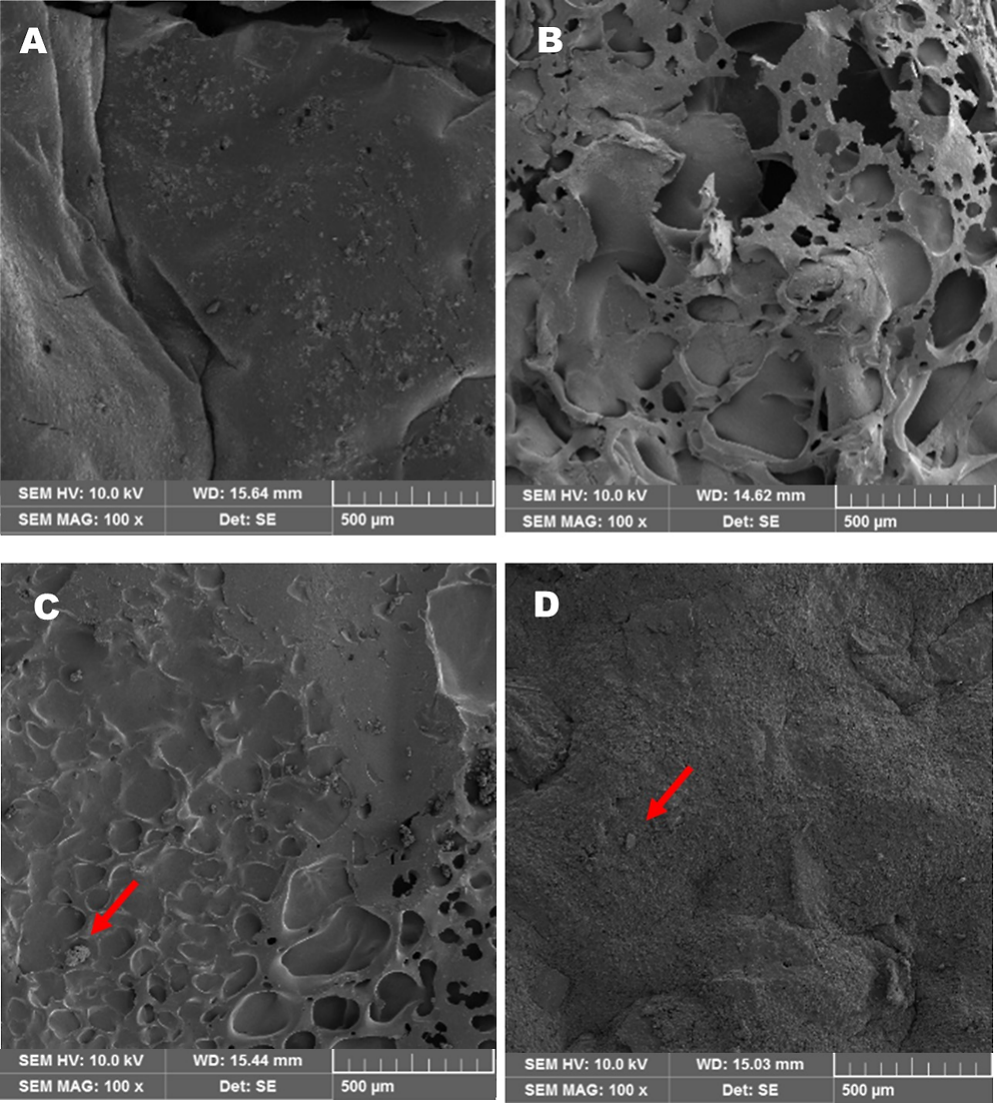
SEM images for the wound dressings. Magnification 100×. (A) Control sample (ZeAg 0%), (B) ZeAg 0.5%, (C) ZeAg 1%, and (D) ZeAg 2%. Red arrows point to the starch granules.

Scanning electron microscopy (SEM) images of the surfaces of the biodegradable wound dressings are presented in Figure 4. The control samples (ZeAg 0%, Figure 4A) and ZeAg 2% samples (Figure 4D) exhibit a more regular and homogeneous surface when compared to the ZeAg 0.5% formulation (Figure 4B). The sample ZeAg 0.5% showed numerous pores across the surface, making the material irregular, which can be attributed to the evaporation of water from the formulation during the sample processing. Due to cavities, this porous structure has a higher capacity for absorbing water and liquids, which is a suitable characteristic for wound dressings used on exudative wounds.

Red arrows highlight the presence of residual starch granules in Figure 4C,D. These granules indicate partial and/or incomplete gelatinization of the starch during thermal processing, which suggests that the higher proportion of ZeAg complexes present in these formulations could result in difficulty in starch gelatinization.

The formulations exhibited a uniform distribution of lignocellulosic fibers derived from BSG, with no apparent aggregates (Figure 4B–D). The distribution of the fibers is a critical factor in evaluating the potential of lignocellulosic fibers as fillers in starch-based matrices. A well-distributed fiber network enhances the interfacial interaction between the fibers and the polymeric matrix, leading to an improvement in the mechanical resistance of the biodegradable dressings.

Wijaya et al.^11^ pointed out that the morphological uniformity of lignocellulosic-derived materials, such as cellulose nanocrystals (CNCs), is influenced by processing methods. In their study, freeze-drying methods led to CNC agglomeration with irregular morphologies, while drop casting prevented particle aggregation, resulting in controllable and uniform rod-like structures.

The distribution of lignocellulosic fibers within the polymeric matrix is critical for enhancing the mechanical properties of biobased composites.^11,38^ The uniformity of the lignocellulosic fibers observed in this study suggests that the processing method was successful in preventing aggregation and promoting the enhancement of the structural integrity of the material.

### 3.3 Thickness, Density, Solubility, and WVP of the Wound Dressings

Table 1 presents the results of the thickness, density, water solubility, and WVP of the samples. The thickness of the biodegradable wound dressings ranged from 2.1 to 3 mm, with no significant differences observed between the samples containing the ZeAg complex. This uniform thickness indicates that the samples were homogeneous, with the BSG fibers well-distributed across the material’s surface, in accordance with SEM findings. The consistent thickness is attributed to precise control of formulation mass during the thermopress process, which ensures that variations in thickness do not affect the mechanical performance.

**Table 1:** Thickness, Density, Solubility, and WVP of the Wound Dressings*

samples	thickness (mm)	density (g·cm^–3^)	solubility (%)	WVP (g·m^–1^·s^–1^·Pa^–1^) × 10^–9^
control	3.066 ± 0.344^a^	0.783 ± 0.082^a^	27.596 ± 2.739^a^	1.747
ZeAg 0.5%	2.776 ± 0.330^a^	1.017 ± 0.300^a^	25.270 ± 1.896^a^	1.813
ZeAg 1%	2.160 ± 0.383^a^	1.094 ± 0.157^a^	30.846 ± 0.895^a^	2.977
ZeAg 2%	2.363 ± 0.208^a^	1.177 ± 0.132^a^	26.875 ± 1.319^a^	1.975

* Samples with different letters in the same column are different (Tukey’s test, *p* < 0.05).

Castanho et al.^39^ developed thermoplastic corn starch composites with BSG and demonstrated that films with the lowest fiber content (1% of BSG) and smaller size (100 mesh) exhibited decreased thickness. However, in this study, the BSG concentration was constant across all formulations; therefore, these parameters did not affect the observed thickness of the materials.

The solubility of the material, which measures the amount of water-soluble substance present, is directly related to its dissolution in water. This test evaluates the integrity of the samples after immersion in water and is influenced by formulation components.^40^ The results showed no significant differences in solubility between the control and ZeAg samples (Table 1).

Despite the lack of a general correlation between solubility and ZeAg concentration, the ZeAg 1% sample exhibited a slightly higher solubility compared to others. This increase in solubility is likely due to the higher surface porosity observed in SEM analysis, which allows more water to penetrate the material.

The addition of ZeAg complexes did not affect the WVP of the dressings (Table 1). García et al.^41^ note that water permeability in polymeric materials is influenced by multiple factors including material thickness, surface integrity, molecular crystallinity, the content of hydrophilic and hydrophobic components, polymer chain mobility, and the presence of plasticizers such as glycerol.

The incorporation of BSG fibers and silver-loaded zeolites into the dressings did not significantly change important properties, such as thickness, density, water solubility, or WVP. A positive balance of properties was observed in the ZeAg 1% sample, which was attributed to its higher surface porosity, which increased the solubility. This indicates that these components are compatible with the starch matrix, ensuring the structural and functional integrity of the materials.

### 3.4 WSI and WAI Indexes

The WSI reflects the degree of starch granule degradation and the severity of thermal treatment as well as the number of water-soluble solids in a formulation. The WAI evaluated the availability of hydrophilic groups (hydroxyls) in the sample, which bind with water molecules leading to gel formation.^42,43^ Considering the application of the materials as biodegradable wound dressings, a high capacity for absorbing the exudate is desirable.

The 0.5% ZeAg formulation exhibited a significantly higher WAI value (4.274 ± 0.495) compared to the other samples (Table 2). SEM images indicate that this formulation has a higher number of pores on its surface, which may have exposed the hydrophilic groups of the polymer, leading to increased water absorption.

**Table 2:** WAI and WSI Indexes of the Biodegradable Wound Dressings^a^

Samples	WAI	WSI
control	2.864 ± 0.098^b^	12.218 ± 1.809^a^
ZeAg 0.5%	4.274 ± 0.495^a^	15.401 ± 4.551^a^
ZeAg 1%	2.341 ± 0.090^b^	18.234 ± 0.314^a^
ZeAg 2%	2.315 ± 0.104^b^	14.061 ± 1.536^a^

a Samples with different letters (a and b) in the same column are different (Tukey’s test, *p* < 0.05).

The WSI values show no significant differences observed between the samples (*p* < 0.05). The literature indicates that adding fibers to a polymeric material typically decreases starch gelatinization because the increased mechanical resistance of the fibers during thermal processing, consequently, reduces water solubility.^44,45^ This trend was also evident in the SEM images, which displayed numerous nongelatinized starch granules.

However, reducing the size of fiber particles by grinding the vegetable fiber (BSG) beforehand helped to maintain the water solubility of the materials. Carvalho et al.^46^ studied the effect of extrusion on mixtures of wheat, rice, and banana flour at temperatures ranging from 60 to 80 °C. Their findings showed that higher temperatures resulted in a higher WSI, due to increased degradation of starch granules.

### 3.5 Mechanical Properties

**Figure 5:**
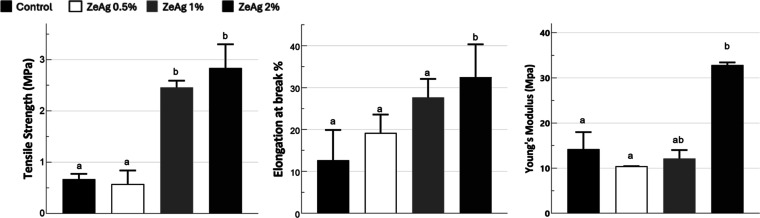
Mechanical properties of the wound dressings.

The maximum tensile strength, elongation at break, and Young’s modulus of the wound dressing materials were evaluated with results shown in Figure 5. Tensile strength represents the maximum amount of stress a material can endure before failure; elongation at break measures the extent to which material can stretch without breaking; and Young’s modulus (*E*) indicates the material’s stiffness. As Young’s modulus value increases, the material becomes stiffer and less prone to deformation.^23^

The results revealed that both tensile strength and elongation at break were significantly enhanced in the ZeAg 1% and ZeAg 2% samples compared to those in the control sample. The addition of BSG and the ZeAg complex in concentrations of 1 and 2% improved both the resistance and flexibility of these samples. Previous studies have confirmed that lignocellulosic fibers can mitigate some of the limitations of starch-based materials.^47,48^

The ZeAg complex positively impacted the mechanical properties of biodegradable wound dressings with increased tensile strength, elongation at break, and Young’s modulus observed in the samples containing higher proportions of ZeAg (2%). This indicates that ZeAg complexes can act as effective reinforcements in starch-based polymeric materials.

The results highlight that ZeAg particles enhance the mechanical performance of biopolymer dressings, making them more suitable for biomedical applications while retaining their antibacterial effect. Additionally, the influence of zeolites on the mechanical properties of starch-based polymers was also studied by Bendahou et al.,^49^ who observed increased tensile strength and Young’s modulus in poly(lactic acid) (PLA) biopolymers with the addition of two types of zeolites.

The improvement in the mechanical properties of the wound dressings in this study can be attributed to the dispersion of the ZeAg particles within the polymeric matrix. The effective interaction between ZeAg, starch, and the BSG through hydrogen bonding likely restricts the mobility of polymeric chains, enhancing the overall material strength and stiffness.^50^ It is common for commercial biopolymeric wound dressings to have suboptimal mechanical properties.^49^ Considering this, the integration of BSG and the ZeAg complex significantly improves their tensile strength and flexibility, making them more resistant to mechanical stress.

### 3.6 Fourier Transform Infrared Spectroscopy

**Figure 6:**
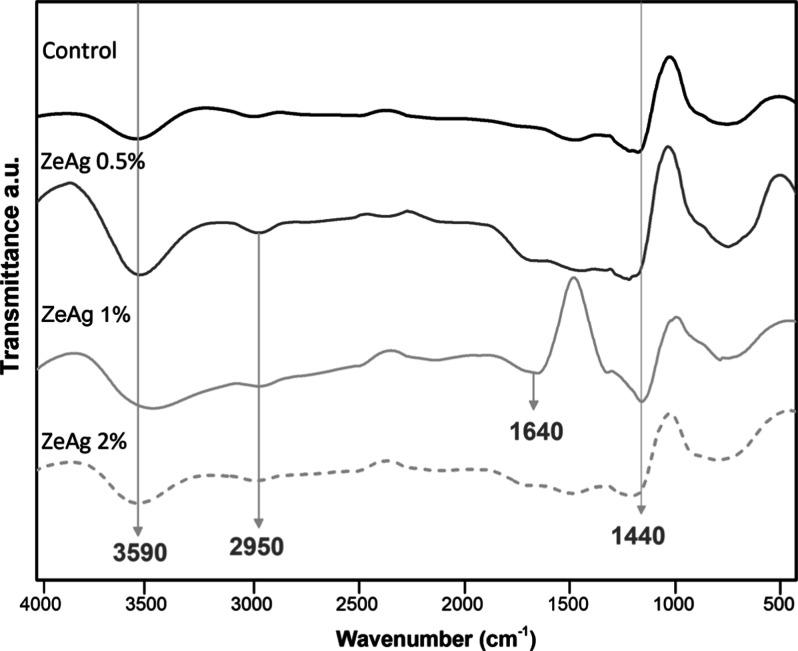
FTIR spectroscopy of the wound dressings.

The structure of the starch-based wound dressings was characterized using Fourier transform infrared (FTIR) spectroscopy. As shown in Figure 6, characteristic bands of the macromolecules and the ZeAg complex were observed.

All spectra in Figure 6 display a broad band at 3590 cm^–1^ in the high-energy region, corresponding to the hydroxyl (−OH) groups of starch, glycerol, and zeolite. According to Lomelí-Ramírez,^51^ the hydroxyl bands of starch at 3300 cm^–1^ are commonly indicative of this type of polysaccharide, which contains glucopyranose rings with a high number of hydroxyl groups.

The small peak observed at 2950 cm^–1^, in the ZeAg 0.5, 1, and 2% samples, corresponds to the symmetric and asymmetric stretching of C–H bonds present in the cellulose and hemicellulose structure from the BSG.^52,53^ This peak is absent in the control formulation due to the lack of BSG in its composition. Additionally, a low-intensity peak observed at 1640 cm^–1^ corresponds to the carbonyl group (C=O) present in the structure of zeolites. The absorption band at 1140 cm^–1^, which can also appear between 1100 and 1200 cm^–1^, represents the asymmetric stretching of C–O–C bonds in the glycosidic linkage between starch and other components.^38^

All formulations showed no additional bands, indicating that no new chemical bonds were formed between the components. This finding is relevant because the occurrence of covalent bonds between the components of the wound dressings could potentially reduce the antimicrobial activity of the complexes.

## 4 Conclusions

Biodegradable wound dressings were successfully developed using starch, BSG, and silver-loaded zeolite (ZeAg) complexes, which demonstrated enhanced functionality. The incorporation of ZeAg significantly improved the mechanical properties while maintaining desirable water absorption and water solubility characteristics, which are crucial for wound dressings. These findings demonstrated the potential of ZeAg complexes as effective reinforcements by combining antimicrobial activity with improved material performance.

This study highlights the potential of agro-industrial byproducts such as BSG to generate sustainable, high-value materials, and cost-effective medical device production. These results provide a foundation for the development of advanced biomaterials, offering innovative candidates for exudative wound care and other biomedical uses.

Translating the ZeAg strategy from the laboratory to clinical settings has considerable potential based on the use of cost-effective zeolites as a natural material and the additional benefit of incorporation of silver ions. The mechanical properties of the material can be adjusted to achieve different wounds, which increases its versatility. This study suggests that with further preclinical and clinical validation, the ZeAg wound dressing could become a valuable alternative to existing treatments.

ZeAg wound dressings have promising potential; however, there are several challenges that must be addressed before they can be widely used in clinical practice. These include the stability of the release of silver ions in real-world conditions, execution of long-term clinical trials to evaluate safety and efficacy, and assessment of the production scalability of the ZeAg dressing to fulfill clinical requirements.

## Data Availability

The data sets generated during and/or analyzed during the current study are available from the corresponding author on reasonable request. Privacy restrictions were imposed on research data in order to register a patent.
